# Study on a horizontal axial flow pump during runaway process with bidirectional operating conditions

**DOI:** 10.1038/s41598-021-01250-1

**Published:** 2021-11-08

**Authors:** Kan Kan, Qingying Zhang, Zhe Xu, Huixiang Chen, Yuan Zheng, Daqing Zhou, Maxima Binama

**Affiliations:** 1 College of Energy and Electrical Engineering, Hohai University, Nanjing, 211100 People’s Republic of China; 2 College of Water Conservancy and Hydropower Engineering, Hohai University, Nanjing, 210098 People’s Republic of China; 3 Nantong Ocean and Coastal Engineering Research Institute, Hohai University, Nanjing, People’s Republic of China; 4 College of Agricultural Science and Engineering, Hohai University, Nanjing, 210098 People’s Republic of China

**Keywords:** Mechanical engineering, Fluid dynamics

## Abstract

The ultra-low head pump stations often have bidirectional demand of water delivery, so there is a risk of runaway accident occurring in both conditions. To analyze the difference of the runaway process under forward runaway condition (FRC) and backward runaway condition (BRC), the whole flow system of a horizontal axial flow pump is considered. The Shear-Stress Transport (SST) *k*–*ω* model is adopted and the volume of fluid (VOF) model is applied to simulate the water surface in the reservoirs. Meanwhile, the torque balance equation is introduced to obtain the real time rotational speed, then the bidirectional runaway process of the pump with the same head is simulated. In addition, the vortex transport equation and swirl number are proposed to reveal the flow characteristics during the runaway process. The results show that the runaway process can be divided into five stages: the drop, braking, rising, convergence and runaway stages, according to the changing law of torque curve. In the rising stage, the pressure difference on the blade surface continues to increase, which contributes to the abnormal torque increase. In this stage, the flow hits the pressure surface (PS) at a faster speed enlarging the pressure on PS, and the flow separation takes place on the suction surface (SS) weakening the pressure on SS. During the convergence and runaway stage, the pulsation amplitude of torque and axial force under FRC is obviously larger than those under BRC. This is because the rotation frequency of the vortex rope is the same as main pressure fluctuation frequency in impeller under FRC, which enhances the pulsation amplitude. Whereas the vortices are broken due to the inhibitive effect from guide vanes under BRC.

## Introduction

The distribution of water resources in China is uneven in time and space due to the special geographical and climatic conditions. Flood and drought disasters occur frequently, and the areas with abundant water resources and high water load are asymmetrical^1^. To solve the problem of water for production and domestic use, China carried out a large number of water diversion, drought and flood prevention projects, represented by the “South–North Water Diversion” strategic project. As a key power support and energy conversion device in this significant project, pump station bears important tasks of water supply and drainage, irrigation allocation, flood prevention and drought prevention, environmental control and river regulation, which also plays a vital role in agricultural production^2^.

When a pump system suddenly stops its normal operations by accident, if the outlet gate fails to cut off the water timely, the water in pump conduit will flow from upstream to downstream, then blades will rotate in the opposite direction under the influence of the backflow^3, 4^. Thereafter, the rotational speed of impeller continues to increase until a stable maximum rotational speed, called runaway speed, is reached. Under runaway condition, the flow pattern inside the conduit will inevitably face violent instability phenomenon, which will easily induce severe pressure pulsations and sharp change of blade stress^5^. Therefore, the research on the transient process of pump system is of great significance for safe and stable operations of pump stations.

A widely applied method for researching the transient process in pump system is one-dimensional method of characteristics (1D-MOC). The MOC was adopted to simulate the water hammer in long-distance water conveyance system at first^6, 7^. Thereafter, a variety of MOC methods were improved to investigate the transient flow for the advantages of high accuracy and robust convergence in hydraulic system^8, 9^. In addition, some scholars also studied the influence of different start-up modes and discharge valve openings on the external characteristics of pumps during the transient process^10, 11^. With the rapid development of modern numerical software, computational fluid dynamics (CFD) has become a useful tool to simulate the evolution law of the internal flow field in pump units^5, 12, 13^. At the same time, three-dimensional (3D) numerical method has been widely applied on the simulation of various transient processes, such as start-up, shut down, runaway process, power off and so on^3, 4, 14^.

Some investigated parameter settings of the relative scholars' literatures on runaway transient process are presented in Table 1. The model test of transient process is dangerous and expensive in most cases, hence, experimental method of steady condition is often used to test the authenticity and accuracy of numerical simulation^15, 16^. For numerical methods, the MOC and 3D simulations differ a lot in modeling and calculation, but only a few scholars combined MOC in pressure pipes with 3D transient simulation in hydraulic units^17, 18^. Simultaneously, CFX and Fluent software became the most widely used CFD components in 3D simulation during transient process owing to their strong adaptability and flexible programmability. To close the control equations with low computational cost and reasonable accuracy, a variety of turbulence models have been widely used in simulations, such as two-equation turbulence models^19–25^, four-equation turbulence model^17, 18, 26^, and the SST based Scale-Adaptive Simulation (SAS) model^15, 16, 27–29^. Additionally, the compressibility of water is not considered for most studies, however, few scholars still adopted the user-defined density function with pressure as an independent variable in their investigations^17, 18, 21^. Considering that the simulation of runaway transient process requires more computing time than general steady simulations, most scholars reduce the number of grid within reasonable limits for fewer computing cost. In most cases, the time-step varied from 1.5 × 10^−4^ to 2 × 10^−3^ s, which makes the runner rotate 0.5–3 degrees per time-step approximately.

**Table 1:** Recapitulation of parameter description of runaway research.

Main author	Turbine	Research method	Runaway head	Dimension	Solver	Turbulence model
Nicolet et al.^30^	Francis turbine	Finite difference method	440 m	1D	SIMSEN	/
Zeng et al.^31^	Pump turbine	MOC	14.5 m	1D	TOPSYS	/
Hosseinimanesh et al.^19^	Francis turbine	Simulation	/	3D	CFX	$\, k - \varepsilon$
Liu et al.^22^	Kaplan turbine	Simulation	1.0 m	3D	Fluent	${\text{RNG}}\;k - \varepsilon$
Fortin et al.^20, 21^	Propeller turbine	Test and Simulation	2.5 m	3D	CFX	$\, k - \varepsilon$
Liu et al.^23^	Axial flow pump	Simulation	2.75 m	3D	Fluent	${\text{Realizable }}k - \varepsilon$
Li et al.^25^	Francis turbine	Simulation	20 m	3D	Fluent	${\text{RNG}}\;k - \varepsilon$
Trivedi et al.^15, 16^	Francis turbine	Test and Simulation	12.26 m	3D	CFX	${\mathrm{SAS}}{\text{-}}{\mathrm{SST}}$
Zhang et al.^17, 18^	Pump turbine	Simulation	10.55 m	1D and 3D	Fluent	$\overline{{\nu^{2} }} - f \,$
Xia et al.^27–29^	Francis turbine	Simulation	/	3D	Fluent	${\mathrm{SAS}}{\text{-}}{\mathrm{SST}}$
Liu et al.^26^	Pump turbine	Simulation	1.78 m	3D	Fluent	$\overline{{\nu^{2} }} - f\;{{\& }}\;{\text{SST}}\;k - \omega$
Kan et al.^32^	Tubular pump	Simulation	2.5 m	3D	Fluent	SST *k *-*ω*
Feng et al.^24^	Bulb turbine	Simulation	5.1 m	3D	CFX	${\text{SST}}\;k - \omega$

All mentioned works above have contributed a lot to parameter setting and research method selection of the runaway transient process within pump and turbine units. Considering the actual water level difference between upstream and downstream, most studies mainly focus on the runaway process of single flow direction, and the reservoirs near the pump system are always ignored for simplification. Therefore, compared to the relative researches on runaway simulation, this paper provides three innovations. Firstly, the runaway process with super low head pump (below 1 m) is considered. Secondly, multiphase flow model was adopted to simulate the free surface between air and water. Lastly, the runaway transient operations under bi-directionally incoming flow are analyzed in this paper.

The remainder of this paper is organized as follows: an entity 3D model of the horizontal axial flow pump is presented and VOF model is introduced in “Numerical methodology” section. In “Results and analysis” section, this paper analyzes the torque and axial force in time and frequency domains, explains the increase of torque and axial force in the rising stage, establishes the link between vortex rope and torque fluctuation amplitude, and exhibits the flow regime in different states. “Conclusion” section summaries the whole work and gives the potential research issues for future research focus.

## Numerical methodology

### Governing equations and turbulence model

The internal flow of a horizontal axial flow pump is governed by the law of mass and momentum conservation. The calculation domain includes upstream and downstream reservoirs with free surfaces, where water and air interact but not interpenetrate. VOF model was developed by Hirt and Nichols^33^ in 1981 to track the interface between immiscible fluids, which can solve highly complex flows with small amount of calculation and simple operation. Thus, the VOF model is suitable for tracking free surface and calculating the volume fraction of water phase. The governing equations of the VOF formulations on multiphase flow are defined as^23^:

The continuity equation

1 $$\frac{\partial \rho }{{\partial t}}{ + }\nabla \cdot \left( {\rho {\varvec{u}}} \right) = 0.$$

The equation of momentum

2 $$\frac{{\partial {\varvec{u}}}}{\partial t} + \left( {{\varvec{u}} \cdot \nabla } \right){\varvec{u}} = - \frac{\nabla p}{\rho } + \nu \nabla^{2} {\varvec{u}} + g.$$

The volume fraction equation

3 $$\frac{{\partial \alpha_{i} }}{\partial t} + {\varvec{u}}\nabla \alpha_{i} = 0$$

where ***u*** is the fluid velocity, $\rho$ is the density, *p* is the static pressure, $\nabla$ is the Hamilton operator, $\nabla^{2}$ is the Laplacian operator, *g* is the gravity acceleration and $\nu$ is kinematic viscosity. $\alpha_{{1}}$ and $\alpha_{{2}}$ are the volume fraction of air and water phase, and $\alpha_{1} + \alpha_{2} = 1$. The density is determined from the following equations:

4 $$\rho = \alpha_{1} \rho_{1} + \alpha_{2} \rho_{2}$$

5 $$\nu = \alpha_{1} \nu_{1} + \alpha_{2} \nu_{2}$$

where $\rho_{{1}}$ and $\nu_{{1}}$ indicates gas phase, $\rho_{{2}}$ and $\nu_{{2}}$ indicates liquid phase.

The SST *k*–*ω* turbulence model, applied to close governing equations in this study, combines the advantages of the *k-ε* turbulence model and *k*–*ω* turbulence model^24^. Specifically, the blending function *F*_1_ is used to determine the selection of *k-ε* model and *k*–*ω* model depending on whether the grid position is close to the boundary layer. Moreover, another blending function *F*_2_ is adopted to modify the definition of the eddy viscosity, in order to consider the transport of turbulent shear stress and avoid overpredicting eddy viscosity^34^. The associated expressions of turbulence kinetic energy, turbulence frequency and eddy viscosity are given in Eq. (6)–(8), respectively.

6 $$\frac{\partial k}{{\partial t}} + u_{j} \frac{\partial k}{{\partial x_{j} }} = P_{k} - \beta^{*} k\omega + \frac{\partial }{{\partial x_{j} }}\left[ {(\nu + \sigma_{k} \nu_{t} )\frac{\partial k}{{\partial x_{j} }}} \right]$$

7 $$\frac{\partial \omega }{{\partial t}} + u_{j} \frac{\partial \omega }{{\partial x_{j} }} = \gamma S^{2} - \beta \omega^{2} + \frac{\partial }{{\partial x_{j} }}\left[ {(\nu + \sigma_{\omega } \nu_{t} )\frac{\partial \omega }{{\partial x_{j} }}} \right] + 2(1 - F_{1} )\sigma_{\omega 2} \frac{1}{\omega }\frac{\partial k}{{\partial x_{i} }}\frac{\partial \omega }{{\partial x_{i} }}$$

8 $$\nu_{t} = \frac{{a_{1} k}}{{\max (a_{1} \omega ,SF_{2} )}}$$

where *k* is turbulence kinetic energy, *ω* is turbulence frequency, *ν*_*t*_ is eddy viscosity, *u*_*j*_ is the velocity component in the *j* direction, *x*_*i*_ and *x*_*j*_ are the Cartesian coordinates components in the *i* and *j* directions, *P*_*k*_ is the turbulence production due to viscous forces, *S* is the invariant measure of the strain rate. All constants are computed by a blend from the corresponding constants of *k-ε* model and *k*–*ω* model via $\gamma = \gamma_{1} F_{1} + \gamma_{2} (1 - F_{1} )$, etc. The constant parameters for this model are: *a*_1_ = 0.31, β* = 0.09, *β*_1_ = 0.075, *γ*_1_ = 0.556, *σ*_*k*1_ = 0.85, *σ*_*ω*1_ = 0.5, *β*_2_ = 0.0828, *γ*_2_ = 0.44, *σ*_*k*2_ = 1, *σ*_*ω*2_ = 0.856.

### Computational domain and boundary condition

**Figure 1:**
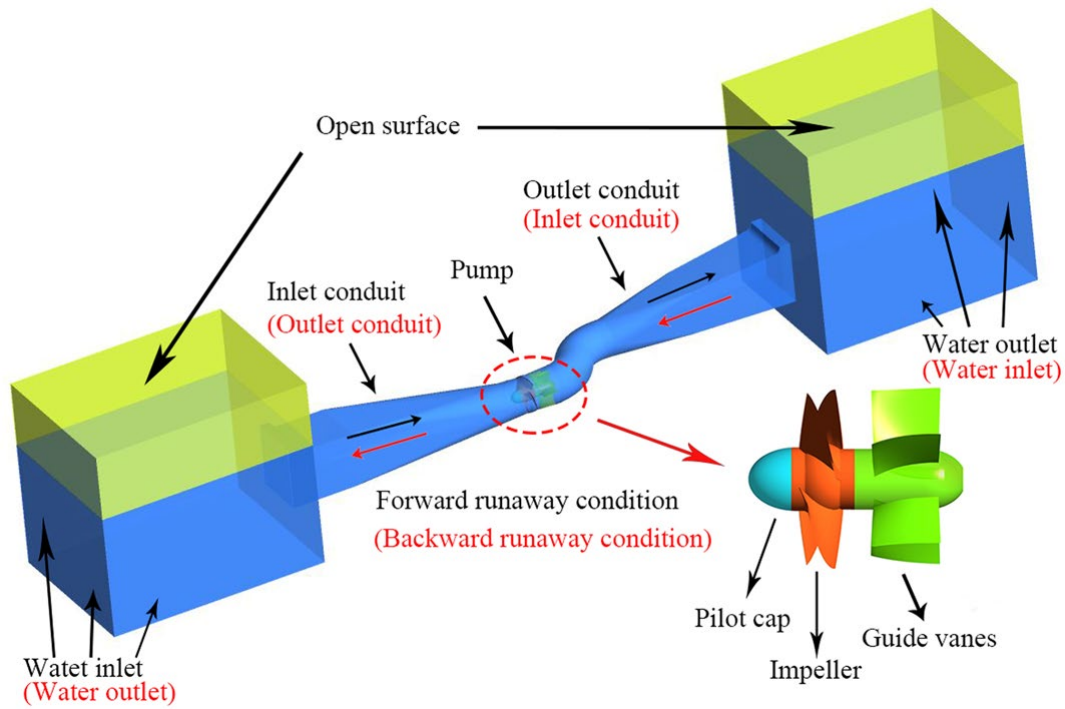
Geometric model of the horizontal axial flow pump system.

A horizontal bidirectional flow shaft extended tubular pump with super low head is investigated in this paper. The characteristic parameters of horizontal axial flow pump under FRC and BRC are shown in Table 2. Figure 1 presents the computational model of the whole pump system including the upstream reservoir, inlet conduit, impellers, guide vanes, outlet conduit and downstream reservoir. In addition, the flow direction under different conditions is identified. The impeller adopts the S-shaped blades for bidirectional flows and the mounting angle of the blades is − 4°. The distance from the inlet conduit to the outlet conduit is 25 m, and the length of the upstream and downstream reservoirs are all 10 m. In addition, the height of the air region is 3.0 m in both reservoirs, and the location of free surface was set according to the actual engineering. As for FRC, the distance from free surface to bottom in upstream and downstream reservoirs are 6.61 m and 5.7 m respectively. In terms of BRC, the distance from free surface to bottom in upstream and downstream reservoirs are 6.61 m and 7.52 m respectively. Moreover, the width of the two reservoirs is 7.5 m.

**Table 2:** Characteristic parameters of horizontal axial flow pump flow system.

Parameters	Value	
	FRC	BRC
Diameter of impeller/m	1.6	
Number of impeller blades/–	4	
Number of guide vanes/–	5	
Blade angle/°	− 8 to 0	
Runaway initial head/m	0.91	
Rotational speed/r/min	170	
Total unit moment of inertia/kg·m^2^	230	
Design discharge/m^3^/s	5	4.5

**Figure 2:**
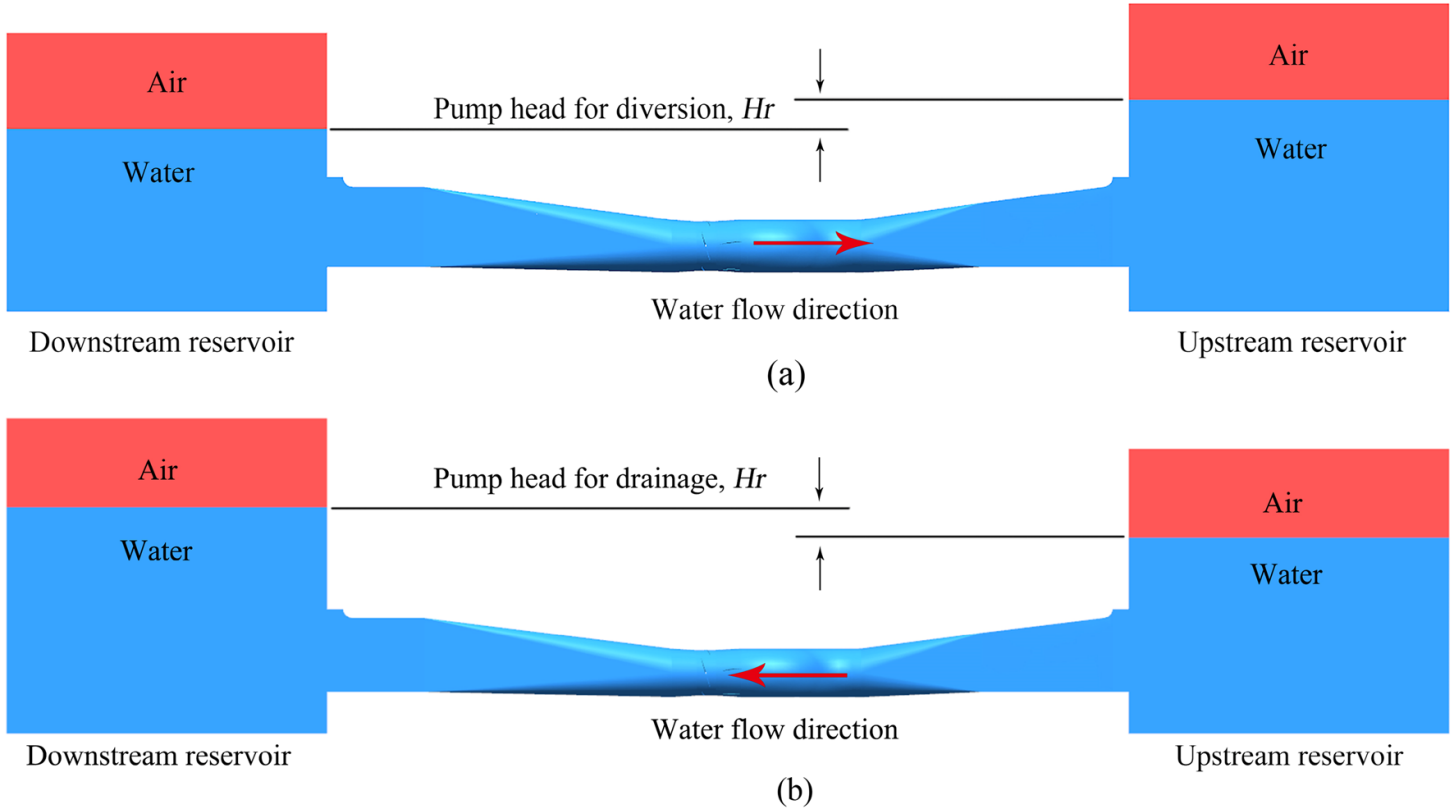
Initial flow field of the calculated domain, (**a**) FRC, (**b**) BRC.

In practical engineering, the ultra-low head pump stations often have bidirectional demand of water delivery. For FRC, the initial flow field of the pump is shown in Fig. 2a, where water flows from downstream reservoir to upstream reservoir and the water height of upstream reservoir is higher than that of downstream reservoir. For BRC, the initial flow field of the horizontal axial flow pump is shown in Fig. 2b, where water flows from upstream reservoir to downstream reservoir and the free surface of downstream reservoir is higher.

In two conditions above, no-slip wall condition was used for all the walls, and the roughness height was set to zero under the assumption that the wall surface is smooth. The boundary conditions of pressure-inlet and pressure-outlet are adopted at the upstream and downstream reservoirs respectively, and the user-defined function (UDF) is applied to let the pressure at the boundary locations change along the water depth, rather than keeping them constant. To be more specific, the hydraulic pressure on the surface of the water is zero, and the hydraulic pressure under water is *ρgh*, where *h* is water depth. What is more, the pressure boundary condition is a typical Dirichlet boundary condition, that is, specify the value of the solution of the differential equation at the boundary, which helps for the simulation convergence.

### Grid generation and sensitivity analysis

In numerical simulation, the quality and quantity of grid have major impact on numerical simulation results accuracy. The ANSYS-ICEM meshing software is used to generate the structured grids in consideration of its good adaptability and high quality of hexahedral structured grid in the flow field. Therefore, O-grids are adopted to divide the inlet and outlet conduit so as to increase the grid density of the boundary layer. In addition, the grids near the wall and free surfaces are encrypted to accurately capture local data.

**Figure 3:**
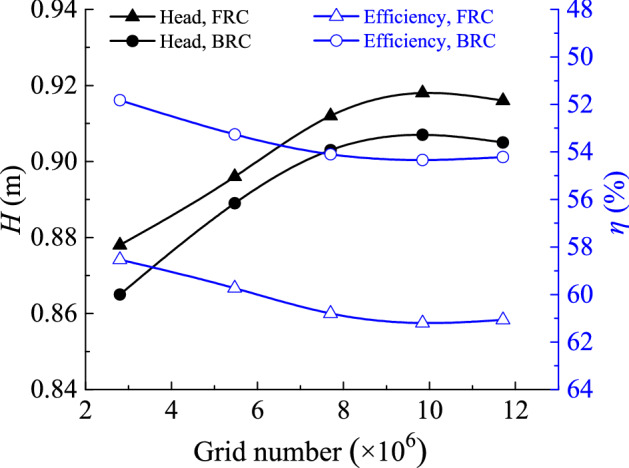
Grid independency test.

**Figure 4:**
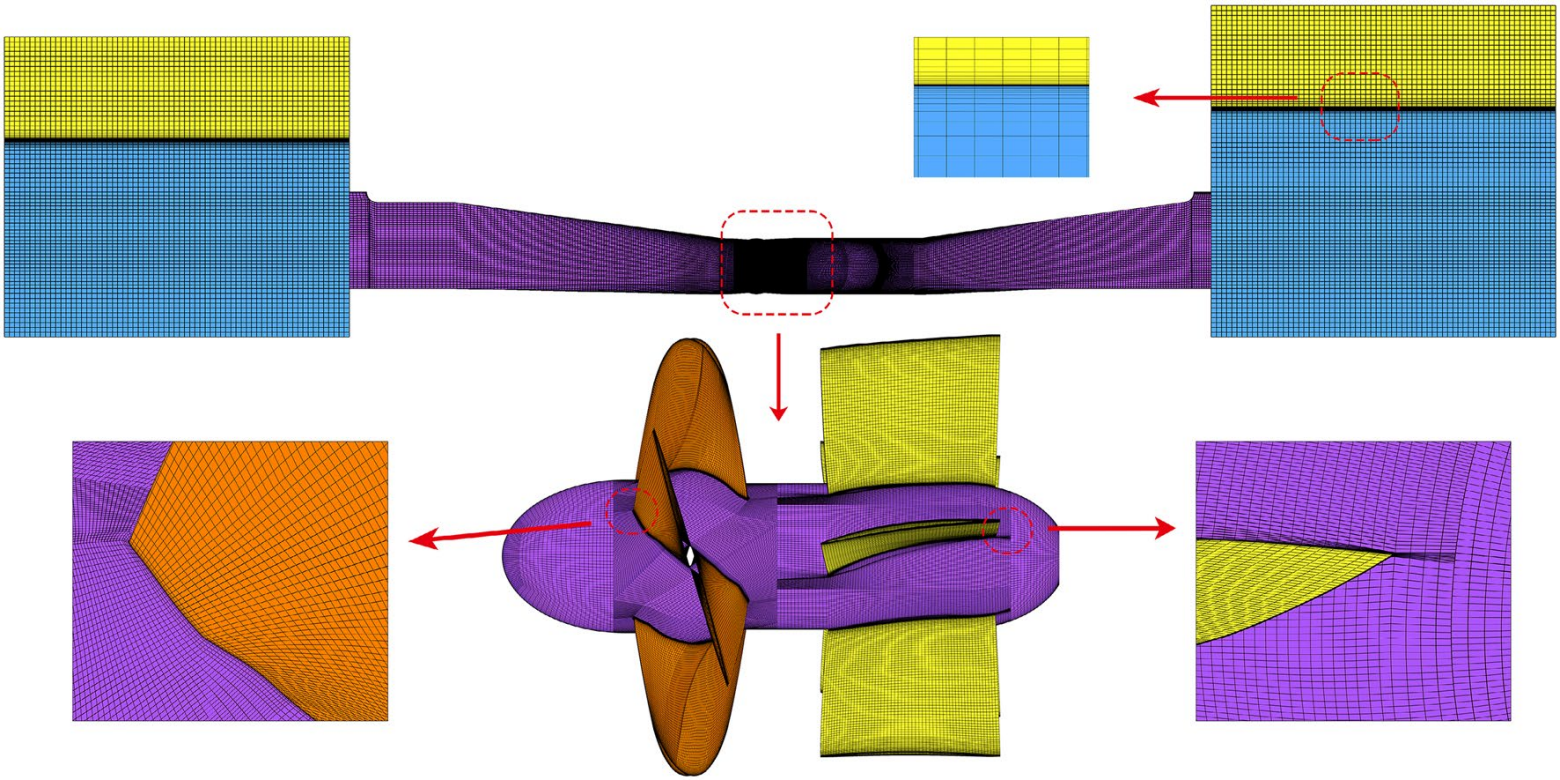
Computational grids of flow components.

The SST *k*–*ω* turbulence model is a near-wall model, which can better predict the wall flow when *y* + is less than 5. In this paper, most grids’ *y* + is less than 1.5, which meets the requirements of SST *k*–*ω* turbulence model. Before the runaway transient simulation, five kinds of grid density are adopted to evaluate the grid independence of the axial flow pump, and the key parameters of the grid are shown in Table 3. Figure 3 shows the effect of grid number on pump head and efficiency under FRC and BRC. When grid number of the whole model came to 7.70 × 10^6^ (Scheme 3), the relative variation ratio of head and efficiency is no more than 0.6%, and the minimum quality is no less than 0.5. After weighing computing resources and grid computing accuracy, scheme 4 is chosen, where the total grid number of flow conduit is 9.84 × 10^6^. Figure 4 presents the computational grids of different flow components.

**Table 3:** Parameter values of different numerical simulation schemes.

Domain	Mesh characteristics	Unit	S1	S2	S3	S4	S5
Inlet conduit	Grid number	/10^5^	6.3	10.4	14.2	19.4	25.9
	Min. angle	/°	32	34	33.5	33.5	33.5
	Grid quality		0.4	0.45	0.5	0.5	0.5
Impeller	Grid number	/10^5^	10.2	14.5	19.6	22.6	26.6
	Min. angle	/°	18	18	27	27	27
	Grid quality		0.3	0.35	0.45	0.45	0.45
Guide vanes	Grid number	/10^5^	6.4	16.3	24.0	32.6	36.0
	Min. angle	/°	27	27	30	30	30
	Grid quality		0.4	0.45	0.5	0.5	0.5
Outlet conduit	Grid number	/10^5^	5.1	13.5	19.2	23.8	28.6
	Min. angle	/°	36	36	35.5	35.5	35.5
	Grid quality		0.35	0.6	0.6	0.6	0.6

### Numerical scheme

In this study, the angular momentum equation is applied to relate rotation speed variation with the torque exerted on the impeller blades^18^. In addition, the user-defined function of FLUENT was introduced to control the torque balance equation of the impeller, that is:

9 $$\frac{dn}{{dt}} = \frac{30}{\pi }\frac{M}{J}$$

where *J* is the total unit moment of inertia, *n* is the rotational speed, *M* is the total torque of the impeller and *t* is the time.

The mechanical friction torque and the rotor wind resistance torque were not considered in the total torque. Then, the rotational speed of every time step is obtained by using:

10 $$n_{i + 1} = n_{i} + \frac{30}{\pi }\frac{M}{J} \times \Delta t$$

where Δ*t* is the time step.

ANSYS Fluent provides a widely used platform for UDF and flexible model selection in fluid numerical simulation. The finite volume method (FVM) with pressure-based solver was adopted to discretize the governing equations. The semi-implicit method for pressure-linked equations-consistent (SIMPLEC) method was applied to the coupling solution of pressure and velocity^23^. A second-order upwind scheme is selected to discretize the convection and diffusion terms. A first-order implicit format is employed to discretize the time term.

In this simulation, the time step is set to 0.001 s to make sure that the convergence criteria of the RSM residuals at each time-step were below a typical criterion of 10^−5^. And the maximum number of the iterations per time step is set to 40. When the maximum runaway speed is reached, the impeller rotation of each time step is about 1.5°.

## Results and analysis

### Validation of performance characteristics

**Figure 5:**
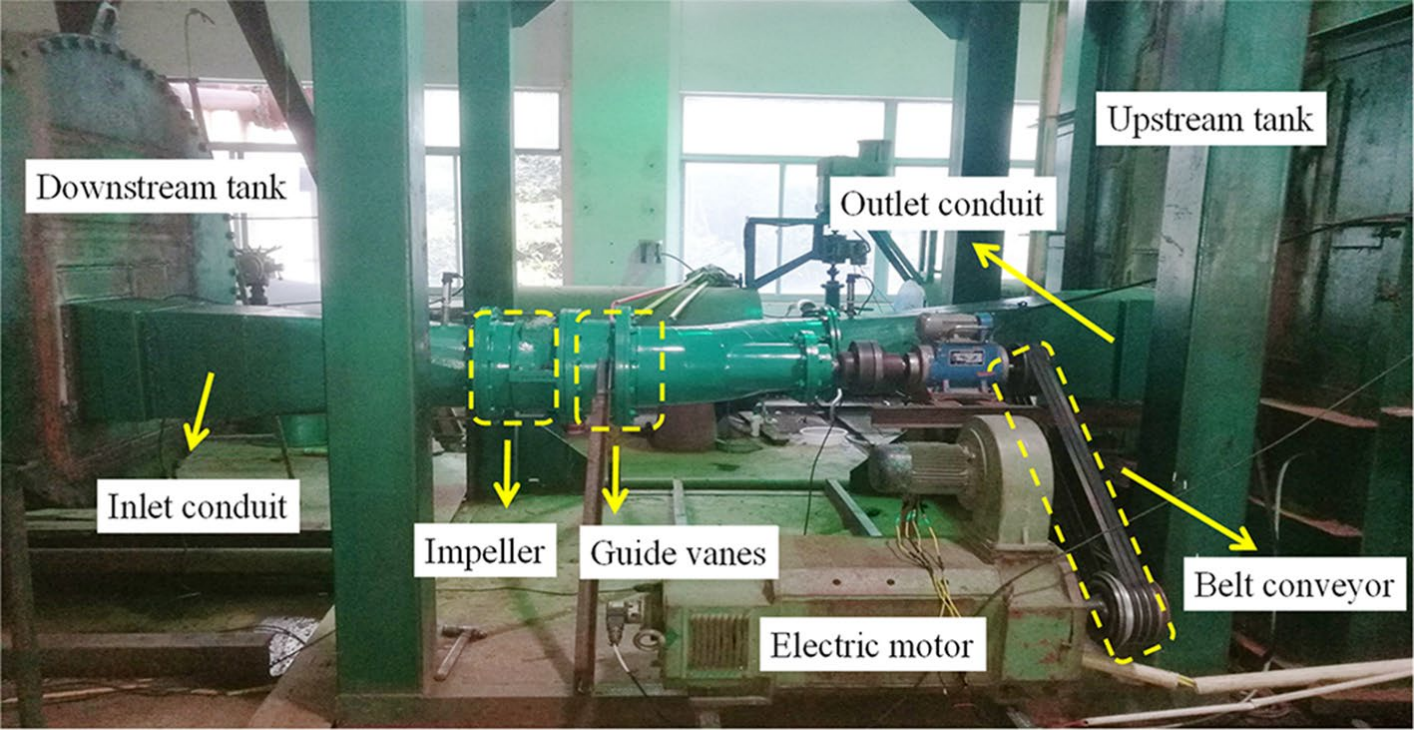
Schematic diagram of model test bench for horizontal axial flow pump with ultra-low head.

In order to verify the simulation results, the model test is required. According to the international standard ISO9906, a model test bench with first grad accuracy is constructed in Hohai University, and the comprehensive uncertainty of test efficiency is less than 0.4%. Figure 5 shows the model test bench, which consists of a downstream tank, an upstream tank, inlet and outlet conduits, an impeller, guide vanes, an electric motor and a belt conveyor among others. Since the demand of bidirectional water delivery in actual engineering, the model test of the ultra-low head pump under bidirectional pump conditions are carried out. Therefore, regardless of the forward or backward pump conditions, at least 15 groups of experimental data under different flow conditions were measured. Additionally, the measured flow points should be reasonably distributed within the range of flow variation. When the test data is stable, the experimental data such as flow rate, pump head, rotation speed and shaft power can be measured.

**Figure 6:**
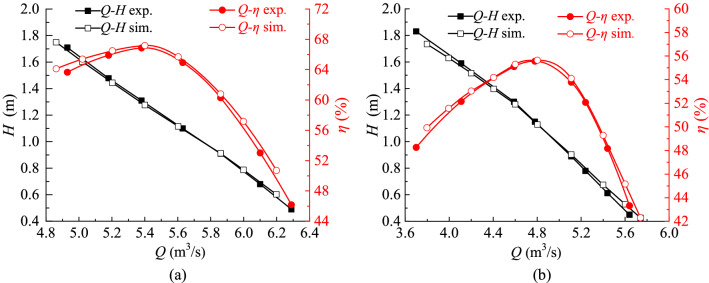
Numerical simulation and test data comparison in terms of pump head and efficiency, (**a**) FRC, (**b**) BRC.

This paper adopts the similarity law to transform the parameters obtained from model test to the prototype ones. Therefore, Fig. 6 compares the head and efficiency of prototype axial flow pump under FRC and BRC. The pump head and efficiency during the simulation can be defined as

11 $$H = \frac{{P_{2} - P_{1} }}{{\rho {\text{g}}}}$$

12 $$\eta = \frac{\rho gQH}{N};N = \frac{nM}{{9.55}}$$

where *H* is the delivery head, *P*_2_ is the total pressure at the outlet of the outlet conduit, *P*_1_ is the total pressure at the inlet of the inlet conduit, *η* is the pump efficiency, *Q* is the flow rate, *N* is the shaft power.

From Fig. 6, the simulation results agree well with experiment results. What is more, in most simulated steady pump conditions, the delivery head error between the experimental and simulated values is less than 3%, and the efficiency error is also less than 3%. There are only two regions where the differences between the simulation and experimental data are more than 3% but no more than 5.5%. For one hand, when the flow rate is lower than 5.0 m^3^/s under FRC and 4.0 m^3^/s under BRC, the experimental head is higher than the simulated one, and the experimental efficiency is lower than the simulated one. In this case, the flow instability at low flow rate may result in the measurement error. For another, when the flow rate is larger than 6.1 m^3^/s under FRC and 5.5 m^3^/s under BRC, the experimental head is lower than the simulated one, and the experimental efficiency is lower than the simulated one. In this situation, the low efficiency under ultra-low head condition means more energy loss, which may enlarge the scale effect and the measurement error. In general, the error between experiment and simulation is acceptable, therefore, the verification of simulation meets the requirement.

### Analysis of the torque and the axial force evolution in time and frequency domains

**Figure 7:**
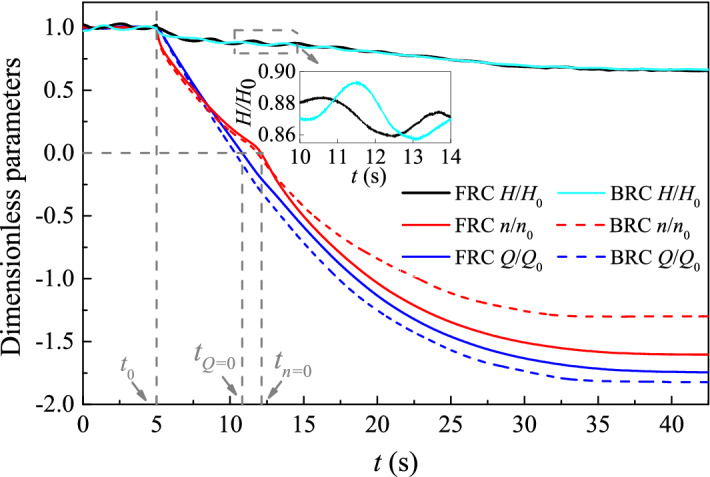
External characteristic curve under FRC and BRC.

**Figure 8:**
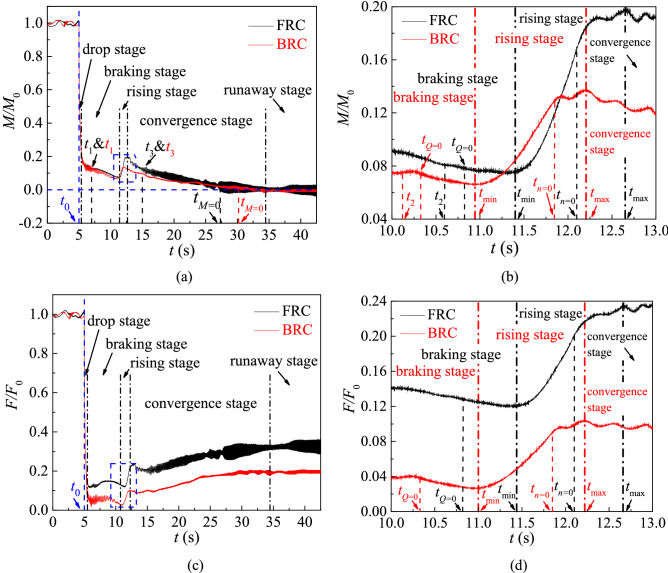
Torque and axial force curve under FRC and BRC, (**a**) torque curves, (**b**) detail torque curves in stage 3, (**c**) axial force, (**d**) detail axial force in stage 3.

To discuss the variation of the dimensionless parameters with time, the external characteristic variables under FRC and BRC are divided by the averaged values at the last impeller rotating period respectively. The *H/H*_0_, *n/n*_0_, *Q/Q*_0_ , *M/M*_0_ and *F/F*_0_ represent the dimensionless head, rotational speed, flow rate, torque and axial force respectively (Figs. 7, 8). The total unsteady simulation time is 42.5 s, and the simulation time of normal pump condition and runaway process are 5 s and 37.5 s respectively. The time *t*_0_ means power-off occurred, the time *t*_1_ = 7 s, *t*_3_ = 15 s and *t*_runaway_ = 42.5 s represent the typical time in the braking, convergence and runaway stage (Fig. 8a), the time *t*_2_ represents the selected time before *t*_*Q*=0_ (Fig. 8b), the time *t*_*Q*=0_ marks the end of the pump station, the time *t*_*n*=0_ marks the start of the turbine condition, the time *t*_*M*=0_ means the torque is equal to zero (Fig. 8a), the time *t*_min_ and *t*_max_ represent the minimum and maximum value respectively during the rising stage (Fig. 8b,d).

From Fig. 7, the rotational speed, water head and flow rate are basically stable in the rated condition (from *t* = 0 s to *t* = 5 s). Once the power-off occurred, the impeller cannot provide power force to drive the flow, therefore the pump head and the flow rate begin to decrease. Thereafter, the change of rotational speed curves is similar under FRC and BRC owing to similar impeller torque from *t* = 5 s to *t*_*Q*=0_. When the impeller rotates in reverse after *t*_*n*=0_, the rotational speed starts to increase as flow rate increases, while the pump head decreases. And the rotational speed under FRC is gradually higher than that under BRC, resulting from the larger flow rate under FRC. When the pump is totally in runaway condition, the head, flow rate and rotating speed are mainly steady.

To detailedly investigate the variation of impeller torque and axial force, the time-domain curves are divided into five stages: drop stage, braking stage, rising stage, convergence stage and runaway stage (Fig. 8). In the drop stage, the torque and axial force of blades drop rapidly due to the motor shutdown. In the braking stage, the flow rate gradually decreases until it drops to zero at *t*_*Q*=0_, which has a braking effect on the blades still rotating in pump mode. In this case, the torque and axial force change at a slower rate than those in the drop stage. Then comes the rising stage, the value of torque and axial force increase from *t*_min_ to *t*_max_. Moreover, *t*_*Q*=0_ is in front of *t*_min_, and *t*_*n*=0_ is between *t*_min_ and *t*_max_, whether it is under FRC or under BRC. As for the convergence stage, the pressure difference between the inlet and outlet drives the impeller to rotate continuously, which makes the torque and axial force tend to be converged. In addition, the torque slowly decreases to zero, and the axial force rises to a stable value in fluctuations. The last stage is the runaway stage, in which the torque and axial force remain stable with slight fluctuations.

Compared the two conditions, the torque and axial force under BRC fluctuate more greatly during the braking stage, which shows that the flow instability is more serious under BRC. In the rising stage, the torque grows by 159% under FRC and 109% under BRC from *t*_min_ to *t*_max_, and the axial force increment under FRC is more than that under BRC. During the convergence and runaway stage, the torque and axial force fluctuate more violently under FRC, which may be related to the complex flow pattern under FRC.

**Figure 9:**
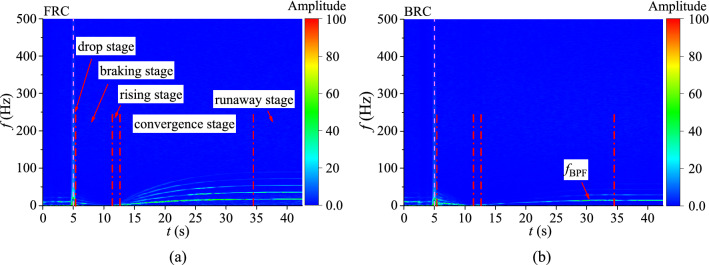
Transient frequency domain diagram of torque fluctuation, (**a**) FRC, (**b**) BRC.

**Figure 10:**
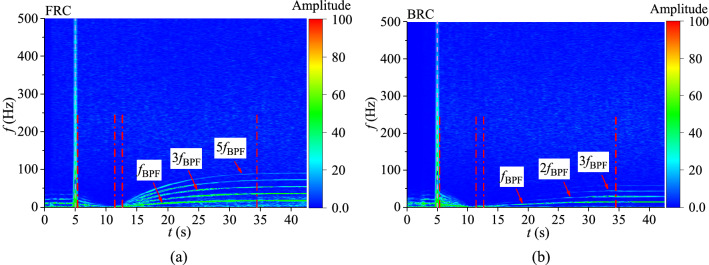
Transient frequency domain diagram of axial force fluctuation, (**a**) FRC, (**b**) BRC.

In order to deeply study the fluctuations of blade torque and axial force under FRC and BRC, the numerical data are processed by short time Fourier transform (STFT) method. The Hanning window function in STFT is selected to avoid spectral leakage and obtain the accurate frequency. The transient characteristics of pulsating frequency and amplitude for torque and axial force are shown in Figs. 9 and 10.

The pulsations of torque and axial force is mainly caused by the pressure fluctuations on blade surface, which is related to the rotor–stator interaction (RSI)^35^. So the frequency is mainly controlled by the blade passing frequency (BPF), which can be expressed as:

13 $$f_{{{\text{BPF}}}} = \frac{{{\text{Z}}n}}{60}$$

where Z is the number of impeller blades and *n* is the rotational speed.

Combined with the speed curve (Fig. 7), it can be confirmed that the BPF under FRC and BRC is different in runaway state. Figure 9 shows the blade torque fluctuation diagram. In case of the braking stage, there is a higher amplitude with low-frequency pulsation of torque under BRC than that under FRC. During the convergence and runaway stage, the main pulsation frequencies of torque under FRC and BRC are BPF and the high harmonics of BPF. However, the amplitudes of the second and third harmonics of the BPF under FRC are much higher than those under BRC, which means the intensity of RSI under FRC is larger than that under BRC. The axial force fluctuation characteristics (Fig. 10) is similar to the torque fluctuation. In the convergence and runaway stage, the torque amplitude of BPF is the largest, followed by 2BPF, 3BPF and so on. Additionally, the main pulsation frequencies of axial force under FRC are BPF and its second, third, fourth and fifth harmonics, whereas the main pulsation frequencies of axial force under BRC are BPF, 2BPF and 3BPF. Therefore, the pulsation amplitude of axial force under FRC is larger than that under BRC, which accounts from the high intensity of pressure pulsation on the blade surface under FRC.

### Pressure pulsation analysis

**Figure 11:**
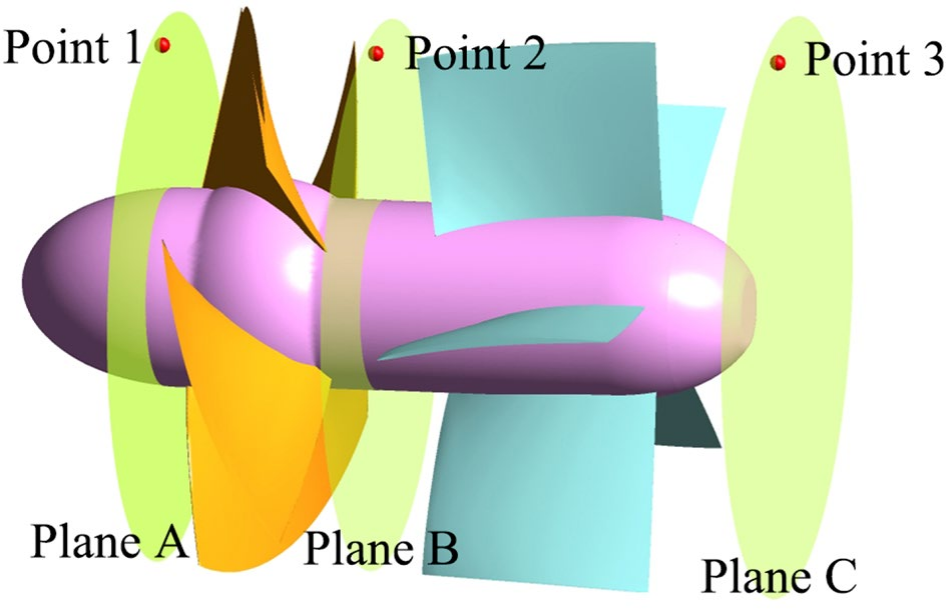
Pressure pulsation monitoring points.

**Figure 12:**
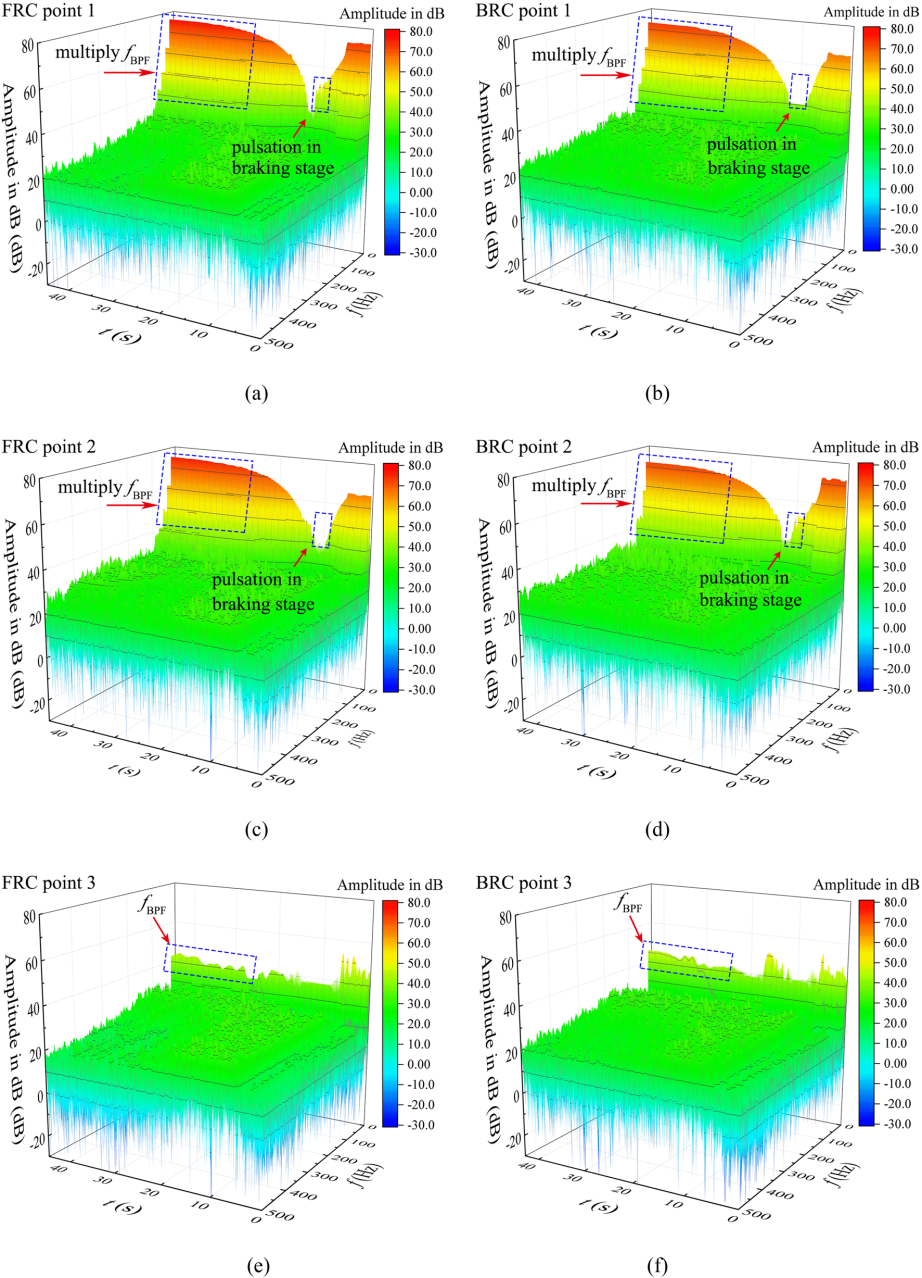
The Transient frequency domain diagram of pressure fluctuation at monitoring points, (**a**) point 1 under FRC, (**b**) point 1 under BRC, (**c**) point 2 under FRC, (**d**) point 2 under BRC, (**e**) point 3 under FRC, (**f**) point 3 under BRC.

The water pressure on the blade surface is the main source of torque value and axial force, so the transient characteristics of the pressure pulsations in pump section are quite important. Figure 11 shows the monitoring planes and points. The monitored pressure data were transformed by STFT in Fig. 12 to obtain a frequency domain of pressure fluctuations.

As a key part of energy conversion components, the impeller changes from a power source to an energy dissipation part during the runaway process. The BPF varies over time, thus the RSI effect during the transient process is different from that in the steady operation. The point 1 under FRC (Fig. 12a) and point 2 under BRC (Fig. 12d) have the similar pulsation characteristics. During the period (0–5 s) before power failure, the pulsation amplitude of the measured pressure at the inflow direction is significantly larger than that at the outflow direction under both FRC and BRC, which maybe is related to the pressure concentration at inlet edge of the blade. During the braking stage, the pressure pulsation amplitude in pump section would be less influenced by rotation speed, because of the limited RSI effect with low rotational speed. Additionally, the high amplitude with low-frequency pulse occurs at the point 1 under FRC and point 2 under BRC, which may result from the flow deterioration at the inflow direction of the impeller. In convergence and runaway stage, the frequency of pressure pulsation is strongly affected by RSI. Therefore, there are several higher harmonics of BPF at point 1 and 2 under FRC and BRC, while the pulsation frequency at point 3 is dominated only by BPF. Furthermore, the pressure pulsation amplitude of points 1 and 2 under FRC is certainly higher than that under BRC, which is the reason why the torque and axial force pulsation amplitude under FRC are obviously large.

### The pressure repartition over the blades during the rising stage

**Figure 13:**
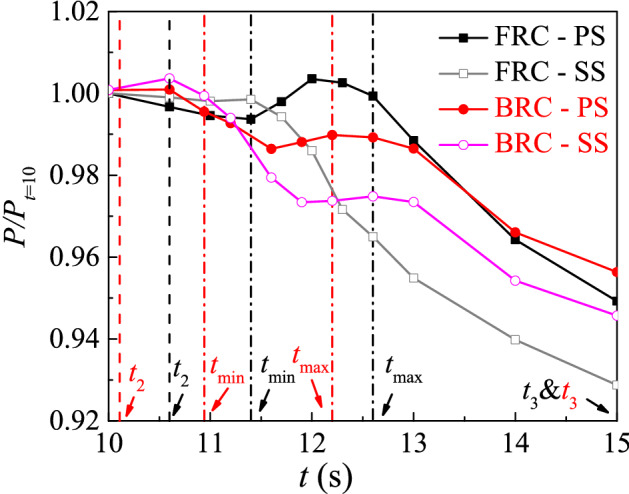
The pressure integral on PS and SS under FRC and BRC.

As we all know, the torque is directly related to the pressure difference on the blade surface. And the pressure integral on the pressure surface (PS) and suction surface (SS) under FRC and BRC are shown in Fig. 13, where *P* represents the pressure integral on PS and SS. To discuss the variation of the dimensionless pressure integral with time from *t* = 10 s to *t* = 15 s (including the rising stage), the pressure integral *P* on PS and SS under FRC and BRC are divided by the value at *t* = 10 s respectively. From Fig. 13, the overall trend of the pressure integral on PS and SS is downward over time. What is more, the pressure integral on PS under FRC and BRC have an increasing part from *t*_min_ to *t*_max_, whereas the pressure integral on SS under FRC and BRC decrease sharply. Therefore, the pressure difference between PS and SS increases from *t*_min_ to *t*_max_, which accounts for the reason why the torque enlarges in the increasing stage (Fig. 8b). Furthermore, the pressure integral difference between PS and SS at *t*_max_ is higher under FRC than that under BRC, which leads to the higher torque under FRC.As a result, the rotational acceleration of impeller is higher at *t*_max_ under FRC than that under BRC. Since the torque under FRC is higher than that under BRC from *t*_max_ to about *t* = 30 s (Fig. 8), the rotational acceleration of impeller is always higher under FRC than that under BRC, which leads to the separation of the dimensionless rotation speed curves after *t*_max_ under FRC and BRC (Fig. 7).

**Figure 14:**
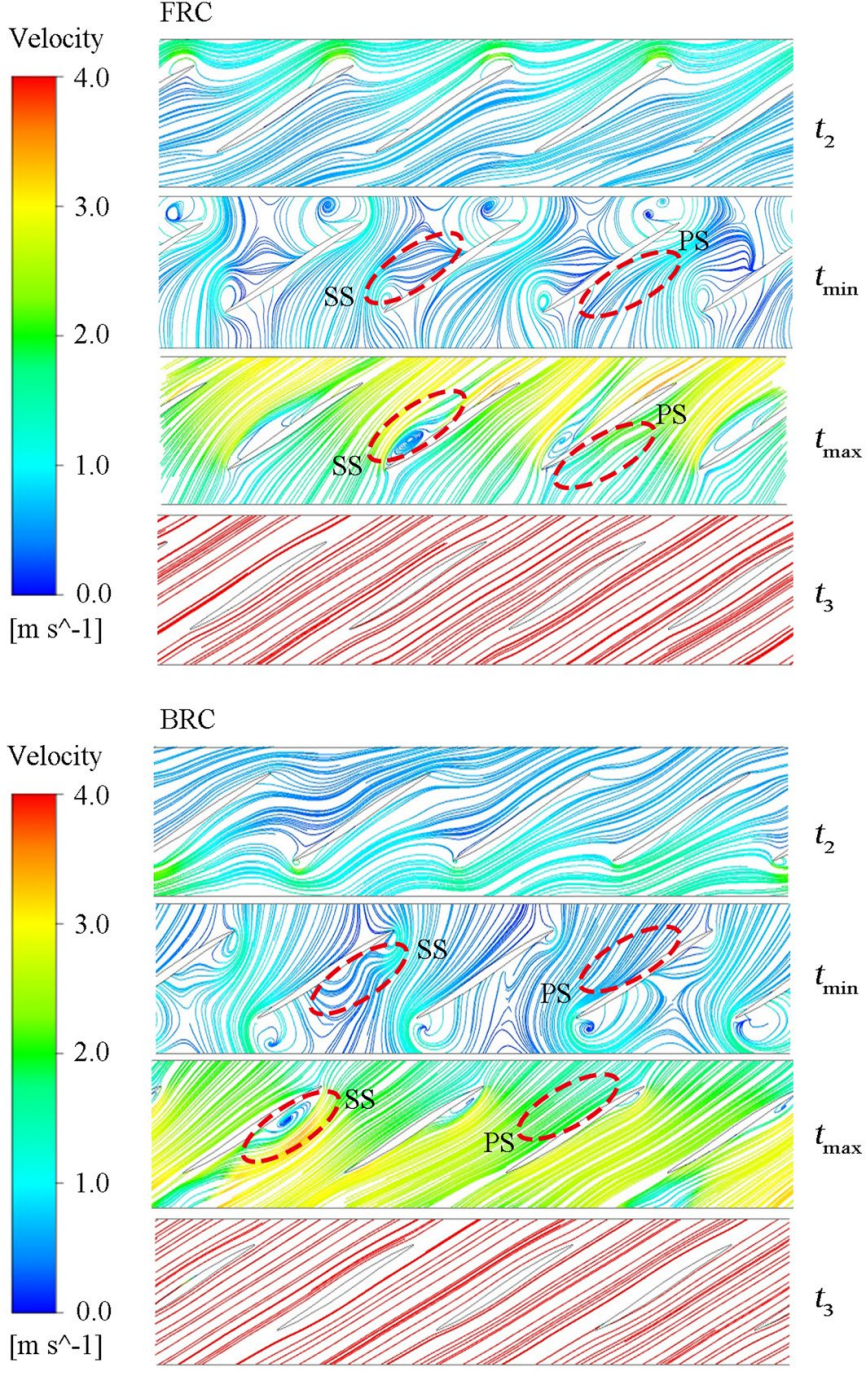
The streamlines on the blade to blade surface at span = 0.5 under FRC and BRC.

In order to deeply research the reason for the pressure repartition over the blades, the streamlines at the middle span are displayed in Fig. 14. When the unit is under pump condition (*t* = *t*_2_), the streamlines inside the impeller tend to be disordered. At *t*_min_, the pump unit begins to experience backward flow, where the streamlines inside the impeller are further deteriorated, and a wide range of low-speed vortices appear. At the *t*_max_ moment, the impeller starts to reverse under the drive of the backflow, which leads to the pressure repartition on the blades. Therefore, the pressure difference on blade surface is larger at *t*_max_ than that at *t*_min_, which contributes to the exist of the rising stage. On the one hand, the flow hits the PS at a faster speed at *t*_max_ than that at *t*_min_, which means the pressure on PS is higher at *t*_max_. On the other hand, the flow separation obviously takes place on SS at *t*_max_, which indicates the pressure on SS is lower at *t*_max_ than that at *t*_min_. In case of *t*_3_, the streamlines are relatively smooth, and there is no inlet impact and flow separation in the impeller.

### Vortex analysis in pump section

In order to research the reason why the torque and axial force oscillate to different degrees, the vortex distribution is taken as an important point to link flow regime with torque and axial force. In addition, the contribution of each component in the vortex transport process is considered. The vortex transport equation is as follows:

14 $$\frac{{{\text{d}}\Omega }}{{{\text{d}}t}} = \left( {\Omega \cdot \nabla } \right)u - \Omega \left( {\nabla \cdot u} \right){ + }\nabla \times f_{i} - \nabla \left( {\frac{1}{\rho }} \right) \times \nabla p + \left( {\nu_{m} + \nu_{t} } \right)\nabla^{2} \omega .$$

In the above equation, the generation of vorticity can be composed of five terms: (1) Vortex stretching term $\left( {\omega \cdot \nabla } \right)u$. (2) Vortex dilatation term $\omega \left( {\nabla \cdot u} \right)$. (3) Physical strength term $\nabla \times f_{i}$, which can be ignored due to the potential of gravity. (4) Baroclinic torque term $\nabla \left( {\frac{1}{\rho }} \right) \times \nabla p$, which is not considered in positive pressure fluid. (5) Viscous dissipation term $\left( {\nu_{m} + \nu_{t} } \right)\nabla^{2} \omega$, which can be ignored in high Reynolds number flows. It is worth noting that vorticity and its components are dominated by positive values.

**Figure 15:**
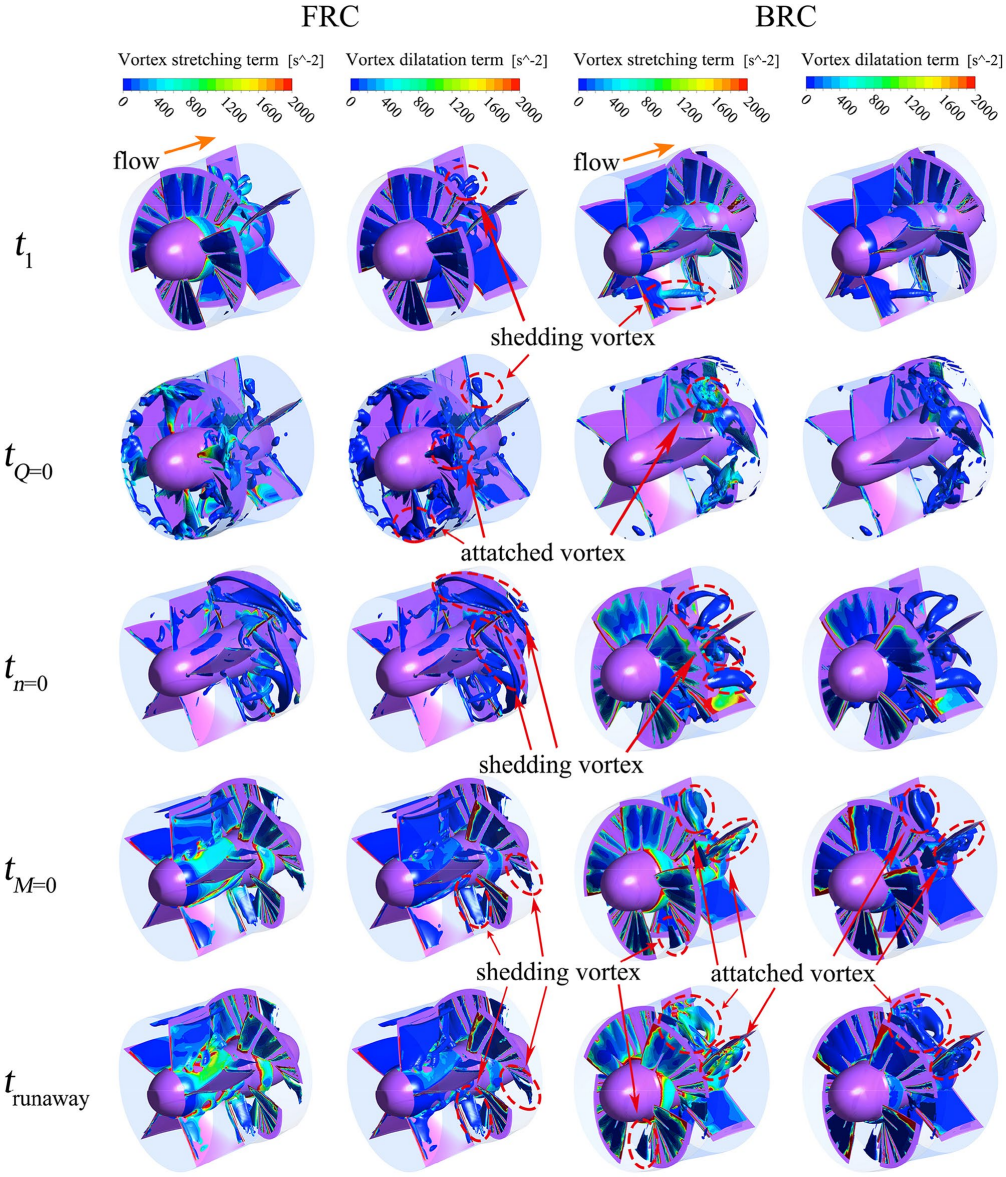
Vortex core at Q = 50/s^2^, and vortex stretching term and vortex dilatation term distribution in impeller.

Figure 15 presents the vortex core, and the vortex stretching term, and the vortex dilatation term distribution under FRC and BRC; where the orange arrows indicate the flow direction, and the Q-criterion is selected to identify strong vortices (actual value of Q is 50/s^2^). It is known that the vortex stretching term is dominant, while the vortex dilatation term is less important under FRC and BRC.

**Figure 16:**
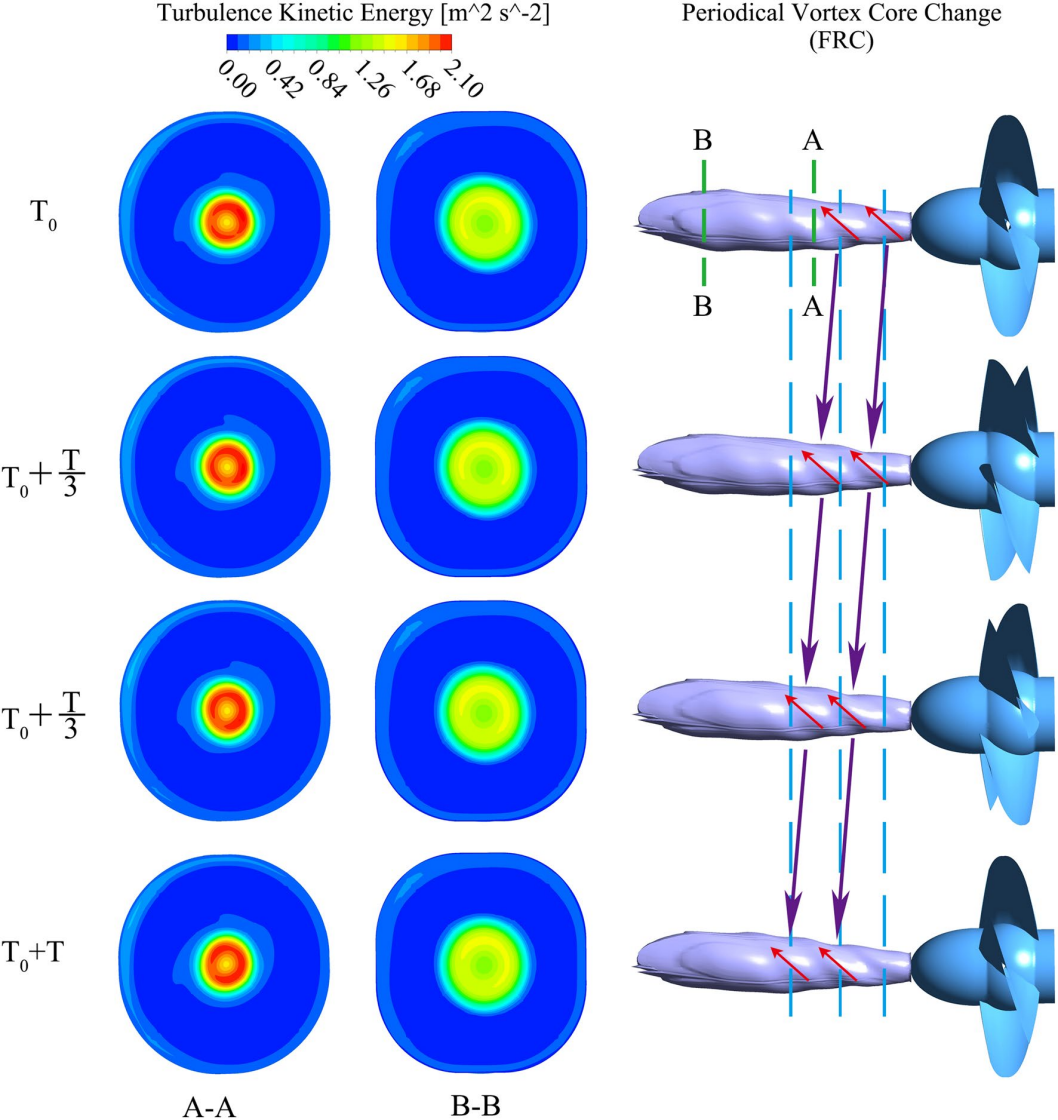
Morphology of periodical vortex rope and TKE distribution during the runaway stage under FRC.

At *t*_1_ (7 s), the shape of shedding vortices is mainly influenced by the conduit in the direction of incoming flow. To be specific, the shedding vortices tend to be annular under FRC owing to the rotational twisted impeller, which disorders the flow and make the vortices bend. While the shedding vortices are inclined to be columnar under BRC due to the fixed guide vanes, which smooths the flow and makes the vortices extend as far as possible. At the moment of *t*_*Q*=0_, the flow pattern in the pump section changes dramatically, and there are attached vortices at the blade inlet, while shedding vortices are dominant at the outlet. At *t*_*n*=0_, since the flow rate is low, the shape of shedding vortices is mainly impacted by the conduit where the vortices locate. Therefore, the vortices are slender around the blades under FRC, while the vortices are short and thick insides the guide vanes under BRC. In case of *t*_*M*=0_ and *t*_runaway_ (42.5 s), the flaky shedding vortices appear at the blade outlet under both FRC and BRC. What’s more, the integrity of vortex rope is under the influence of the location of the guide vanes and the impeller, i.e., the vortex rope maintains intact under FRC but dispersed under BRC. In this situation, the axisymmetrical vortex rope rotates periodically (Fig. 16), which means the velocity gradient within the impeller change periodically under FRC. In addition, the rotation frequency of vortex rope is the same as the main frequency of the pressure pulsation in impeller (i.e. 18.1 Hz), which indicates that the vortex rope can enforce the pulsation of torque and axial force under FRC. Thus, the amplitude of the torque and axial force under FRC is larger than that under BRC.

Figure 16 indicates the periodic vortex rope and turbulence kinetic energy (TKE) distribution during the runaway stage under FRC. There are several whorls on the vortex rope surface, among which the three whorls in the closest vicinities to the impeller are clearly visible. After a period of rotation, the phase of the surface whorls of vortex rope is consistent, and the first and second whorls have developed to the second and third whorls. And the rotation frequency of vortex rope is the BPF. As for the TKE distribution, it clearly exhibits the energy dissipation distribution under the influence of the vortices and rotation blades. For one thing, the TKE at the center section is large, which is consistent with the vortex rope distribution. For another, the TKE of section A is stronger than that at section B, which reveals that the TKE decreases with the increase of distance from the blades.

### Flow pattern analysis in conduit

The flow patterns under the two investigated conditions are different due to differences in terms of respective vortex distribution modes. To describe the swirl level quantitatively in the runaway process, the swirl number (*S*_*w*_) is introduced as follows^36^:

15 $$S_{w} = \frac{{\int_{0}^{R} {U_{x} U_{t} r^{2} {\text{d}}r} }}{{R\int_{0}^{R} {U_{x}^{2} r{\text{d}}r} }}$$

where *U*_*x*_ is the axial velocity, *U*_*t*_ is tangential velocity and *R* is the hydraulic radius, representing the impeller radius.

**Figure 17:**
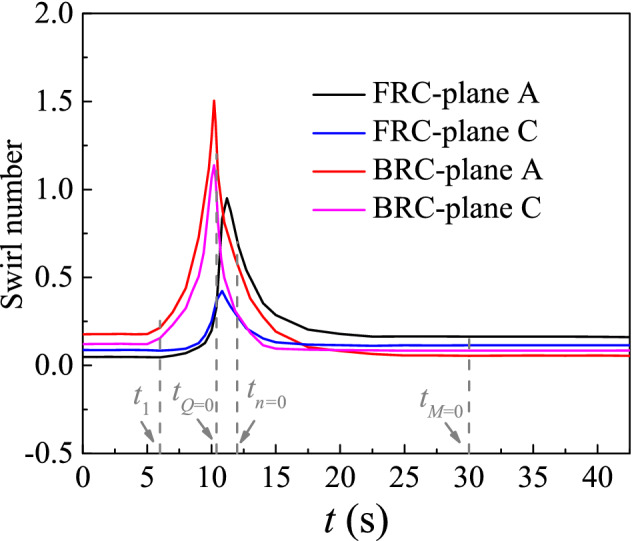
Swirl number of the monitoring planes under FRC and BRC.

**Figure 18:**
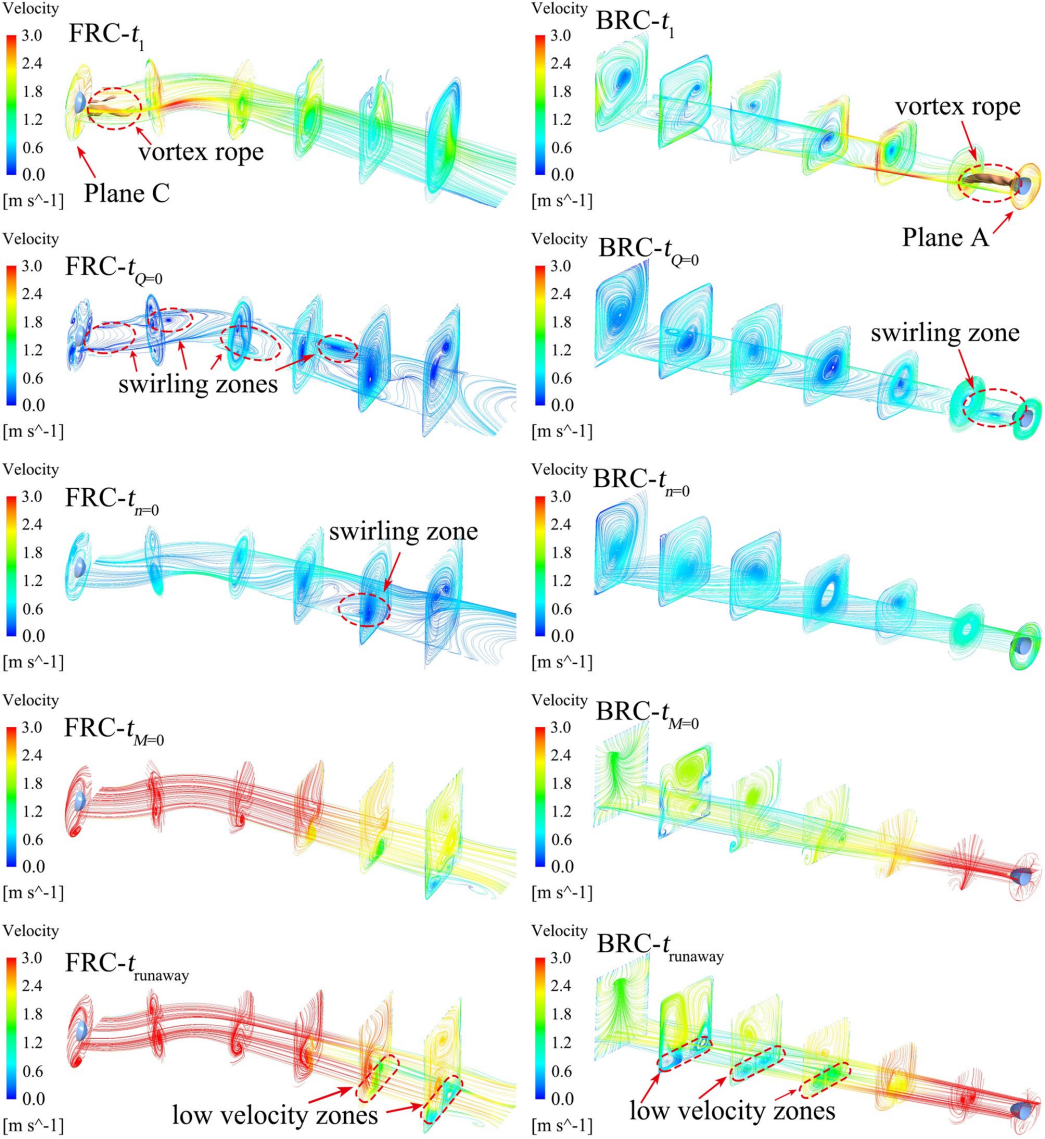
Streamline and vortex core distribution of outlet conduit under FRC and BRC.

**Figure 19:**
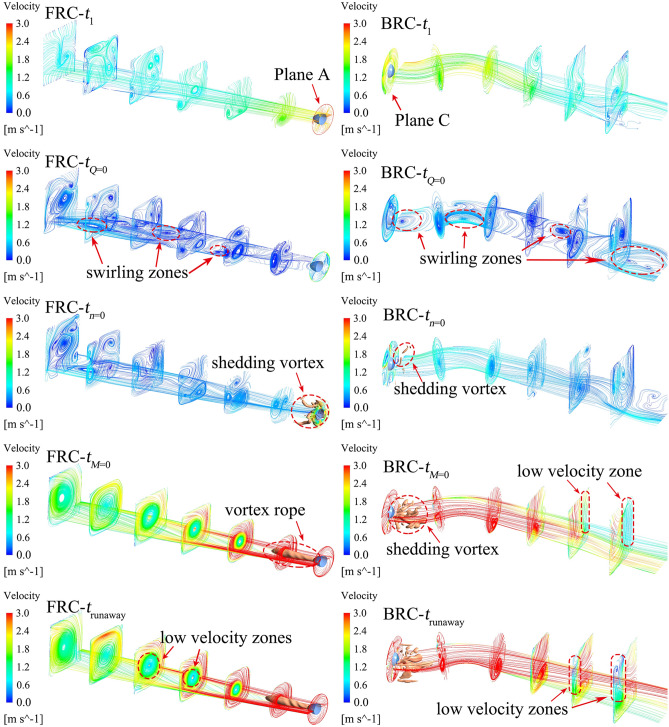
Streamline and vortex core distribution of inlet conduit under FRC and BRC.

Figure 17 represents the swirl number (*S*_*w*_) of the monitoring planes. And the value of *S*_*w*_ is mainly related to the shedding vortices (Fig. 15) and the wide swirl zones (Fig. 18 and 19). Figure 18 displays the streamlines and vortex core distributions (actual value of Q is 50/s^2^) in the outlet conduit under FRC and BRC. At *t*_1_ (7 s), there exists a vortex rope at the outlet of the pump section. At this time, the *S*_*w*_ of plane A under BRC is obviously higher than the *S*_*w*_ of plane C under FRC, owing to the suppressed tangential velocity near the guide vanes of plane C under FRC. At *t*_*Q*=0_, the flow state near the pump section is seriously unstable and the velocity is lower than that in other time. What is more, the rapid rising *S*_*w*_ leads to the vortices breakdown in the impeller section (Fig. 15). At *t*_runaway_, there is a low velocity zone at the bottom of the flow passage, which may be related to the diffusion form of flow passage.

Figure 19 shows the flow pattern of the inlet conduit under FRC and BRC. At *t*_1_, the *S*_*w*_ of plane C is slightly higher than that of plane A (FRC), because the water flow from plane A to plane C and the tangential velocity is higher at the outlet direction of the pump section. At *t*_*n*=0_, the flow starts to reverse and pushes the shedding vortices at the pump section to the inlet conduit. In case of *t*_*M*=0_ and *t*_runaway_, the vortex rope remains intact under FRC, owing to the low tangential velocity in the inlet conduit. At *t*_runaway_, the steady *S*_*w*_ indicates the stable flow pattern. Firstly, the steady water level difference between upstream and downstream leads to the stable flow rate and rotation speed. Secondly, the stable velocity gradient contributes to the stable distribution of vortex rope and shedding vortices. Thirdly, the RSI in a dynamic equilibrium helps to structure the periotic pulsation of pressure, torque and axial force.

## Conclusion

In this paper, the transient characteristics of a horizontal axial flow pump with ultra-low head under FRC and BRC are simulated and analyzed. The VOF model is adopted to take into account the influence of free surface of upstream and downstream reservoirs, where the rotational speed is solved by using the torque balance equation. Two aspects pertaining to differences in pump operating characteristics under FRC and BRC are emphatically analyzed. For one hand, the rising stage characteristics and associated differences between the two investigated conditions. For another, the reason behind large torque fluctuation amplitudes in the runaway stage for FRC. The conclusions are as follows:

1. The time-domain curves of torque and axial force are divided into five stages: drop stage, braking stage, rising stage, convergence stage and runaway stage. And the pulsations frequency of torque and axial force is mainly controlled by the BPF.
2. In the rising stage of torque curve, that is, the braking condition, the pressure difference on the blade surface continues to increase, which serves a direct reason for the endured abnormal torque increase. Meanwhile, the pressure difference on the blade surface under FRC is larger than that under BRC, therefore, the increase of the torque is more serious under FRC than BRC.
3. When the unit is in runaway state, the torque pulsation amplitude under FRC is obviously larger than that under BRC. This is because the rotation frequency of the vortex rope is the same as pressure fluctuation frequency under FRC, and then amplitude of the pressure fluctuation is enhanced. Thus, the amplitude of torque and axial force is strengthened in the runaway stage under FRC. However, the vortex rope is broken due to the inhibitive effect from guide vanes under BRC, which fails to enhance the pulsation amplitude of the pressure, torque and axial force.
4. At *t*_*n*=0_, the special flow pattern products shedding vortices of different shapes, namely, the shedding vortices are slender in impeller under FRC, while they are columnar in the guide vanes under BRC.
5. More comparative research about runaway process with different head and pump types can be carried out as the next step, and the characteristics of parameters in each stage should be different. Additionally, it is necessary to further investigate how to effectively control the flow and structural instability according to the flow characteristics under the runaway condition.

## List of symbols

*f*: Frequency (Hz)
*f*_*i*_: External body force term (N)
*g*: Gravity acceleration (m/s^2^)
Z: The number of the impeller blades
*R*: Radius of the impeller (m)
Z: The number of impeller blades
*H*: Delivery head (m)
*H*_0_: Initial head (m)
*J*: Total unit moment of inertia (kg·m^2^)
*M*: Total torque of the impeller (kN·m)
*M*_0_: Initial total torque of the impeller (kN m)
*N*: Shaft power of the pump (W)
*F*: Axial force of the impeller (kN)
*F*_0_: Initial axial force of the impeller (kN)
*F*_1_, *F*_2_: The blending function of SST *k*–*ω* turbulence model
*n*: Rotational speed (r/min)
*n*_0_: Initial rotational speed (r/min)
*Q*: Flow rate (m^3^/s)
*Q*_0_: Initial flow rate (m^3^/s)
*P*: Pressure integral (N)
*P*_1_: Total pressure at the inlet of the inlet conduit (Pa)
*P*_2_: Total pressure at the outlet of the outlet conduit (Pa)
*P*_*k*_: The turbulence production due to viscous forces (m^2^/s^3^)
*p*: Static pressure (Pa)
*t*: Time (s)
Δ*t*: Time-step (s)
*η*: Efficiency (%)
*k*: Turbulence kinetic energy (m^2^/s^2^)
*ω*: Turbulence frequency (/s)
*α*: Phasic volume fraction
***u***: Velocity (m/s)
*u*_*j*_: Velocity component (m/s)
*U*_*x*_: The axial velocity (m/s)
*U*_*t*_: The tangential velocity (m/s)
*x*_*i*_*, x*_*j*_: Cartesian coordinates components (m)
*S*: The invariant measure of the strain rate (/s)
*S*_*w*_: Swirl number
*Ω*: Vorticity (/s)
*ρ*: Density (kg/m^3^)
*ν*: Kinematic viscosity (m^2^/s)
*ν*_m_: Mixture kinematic viscosity (m^2^/s)
*ν*_t_: Eddy viscosity (m^2^/s)
$$\nabla$$: Hamilton operator
$$\nabla^{2}$$: Laplacian operator

## Abbreviations

1-D: One-dimensional
3-D: Three-dimensional
BPF: Blade passing frequency
BRC: Backward runaway condition
CFD: Computational fluid dynamics
FRC: Forward runaway condition
FVM: Finite volume method
MOC: Method of characteristics
PS: Pressure surface
RANS: Reynolds averaged Navier–Stokes
RSI: Rotor–stator interaction
SS: Suction surface
SST: Shear-stress transport
STFT: Short-time Fourier transform
SIMPLEC: Pressure-linked equation-consistent
TKE: Turbulence kinetic energy
UDF: User defined function
VOF: Volume of fluids
